# The Essential Role of Open Data and Software for the Future of Ultrasound-Based Neuronavigation

**DOI:** 10.3389/fonc.2020.619274

**Published:** 2021-02-02

**Authors:** Ingerid Reinertsen, D. Louis Collins, Simon Drouin

**Affiliations:** ^1^Department of Health Research, SINTEF Digital, Trondheim, Norway; ^2^Department of Circulation and Medical Imaging, Norwegian University of Science and Technology (NTNU), Trondheim, Norway; ^3^NIST Laboratory, McConnell Brain Imaging Center, Montreal Neurological Institute and Hospital, McGill University, Montréal, QC, Canada; ^4^Laboratoire Multimédia, École de Technologie Supérieure, Montréal, QC, Canada

**Keywords:** ultrasound, neurosurgical navigation, open source software, machine learning, open data

## Abstract

With the recent developments in machine learning and modern graphics processing units (GPUs), there is a marked shift in the way intra-operative ultrasound (iUS) images can be processed and presented during surgery. Real-time processing of images to highlight important anatomical structures combined with in-situ display, has the potential to greatly facilitate the acquisition and interpretation of iUS images when guiding an operation. In order to take full advantage of the recent advances in machine learning, large amounts of high-quality annotated training data are necessary to develop and validate the algorithms. To ensure efficient collection of a sufficient number of patient images and external validity of the models, training data should be collected at several centers by different neurosurgeons, and stored in a standard format directly compatible with the most commonly used machine learning toolkits and libraries. In this paper, we argue that such effort to collect and organize large-scale multi-center datasets should be based on common open source software and databases. We first describe the development of existing open-source ultrasound based neuronavigation systems and how these systems have contributed to enhanced neurosurgical guidance over the last 15 years. We review the impact of the large number of projects worldwide that have benefited from the publicly available datasets “Brain Images of Tumors for Evaluation” (BITE) and “Retrospective evaluation of Cerebral Tumors” (RESECT) that include MR and US data from brain tumor cases. We also describe the need for continuous data collection and how this effort can be organized through the use of a well-adapted and user-friendly open-source software platform that integrates both continually improved guidance and automated data collection functionalities.

## Introduction

Ultrasound (US) is the most affordable and least invasive modality for intra-operative imaging of the brain. It is portable, flexible and provides real time imaging whenever needed during the procedure. The progress of surgery can therefore be closely monitored without major delays or interruptions in the workflow. When combined with neuronavigation, US images can be acquired directly in the patient’s frame of reference and are therefore independent of any image-to-patient registration. Consequently, the most recently acquired US images provide the most accurate and up-to-date information about the patient’s anatomy at any given time (1. Despite these advantages, US-guided neurosurgery is still not widely adopted in routine clinical practice. The availability of fully integrated US solutions for neuronavigation systems remains limited. Only a few specialized centers can afford costly extensions to their navigation systems such as BrainLab™’s US navigation module ^1^, and these solutions only provide a simple display of live US images in the context of preoperative scans. All other neurosurgeons who use US imaging must rely on real-time 2D US displayed on the monitor of the scanner, separate from the neuronavigation system, which makes it difficult to map the information on the scans back to the patient. Furthermore, US images present unfamiliar contrast, noise, and artefacts, which further limits its clinical usefulness.

To address these limitations, more research is needed on the processing, visualization, and integration of US images with existing navigation systems. To fuel the next wave of research on those topics, large amounts of data gathered from real surgical cases is needed. Over the past 15 years, our respective research groups have developed world leading expertise in the acquisition, processing and display of intra-operative ultrasound (iUS) data through the development of open source software platforms CustusX (2) and Ibis Neuronav (3). Despite the availability of such platforms, the efforts to collect and distribute the resulting data have remained limited to one center. However, recent efforts from the open source community have enabled the standardization of interfaces between research software and proprietary medical equipment installed in different centers [e.g. the Plus Toolkit (4)], and the interoperability between existing software platforms for the acquisition and processing of US images (e.g., IGSIO^2^). These recent developments open the possibility for multi-center efforts for large-scale data collection. Such efforts would provide researchers with the quantity and variability of ultrasound data required for the technical developments needed for ultrasound to become a truly widespread and useful tool in neurosurgery.

In this paper, we describe the development of existing open-source ultrasound based neuronavigation systems and how these systems have contributed to enhanced neurosurgical guidance over the last 15 years. We also review the impact of the large number of projects worldwide that have benefited from the existing publicly available datasets “Brain Images of Tumors for Evaluation” BITE (5) and “Retrospective evaluation of Cerebral Tumors” (RESECT) (6) that include MR and US data from brain tumor cases. Finally, we describe the increasing need for collecting large amounts of data to meet the requirements of recently developed machine learning algorithms, the possible organization of data collection through the use of an open-source software platform and the potential for new developments in ultrasound guided neurosurgery.

## Materials and Methods

In this section, we describe existing open source systems and how, together with the public datasets they have helped to acquire, these systems have contributed to the advancement of iUS-based navigation in neurosurgery.

### Existing Systems

Commercial image-guided neurosurgery (IGNS) systems such as Medtronics™, BrainLab™, Stryker™, Synaptive™, and others are widely used for surgical planning and guidance worldwide. These systems are built for routine clinical use and their user interfaces are designed for easy use by surgeons and clinical staff. However, these systems are largely closed and are not built for systematic data collection for research purposes. In general, the mechanism provided to export data gathered during an operation is restricted to a certain portion of the information available and can only be done after the end of the procedure. In some cases, a software development kit allows for third parties to develop application that can capture real-time data such as 3D pose of certain tracked surgical tools using a separate computer (e.g., Medtronic’s StealthLink™ or BrainLab™’s support for OpenIGTLink). However, these development kits are either costly or require specific research agreements between the hospital and the manufacturer, and their interfaces tend to change frequently, which is likely to disrupt long-term or multi-center research projects.

To address the limitations of the commercial systems, our respective research groups have developed world leading expertise in the acquisition of various types of intraoperative data through the development of open source software platforms. The two systems, Ibis Neuronav, developed by the Montreal group, and CustusX developed by the Trondheim group are described in the following sections.

#### Ibis Neuronav

**Figure 1:**
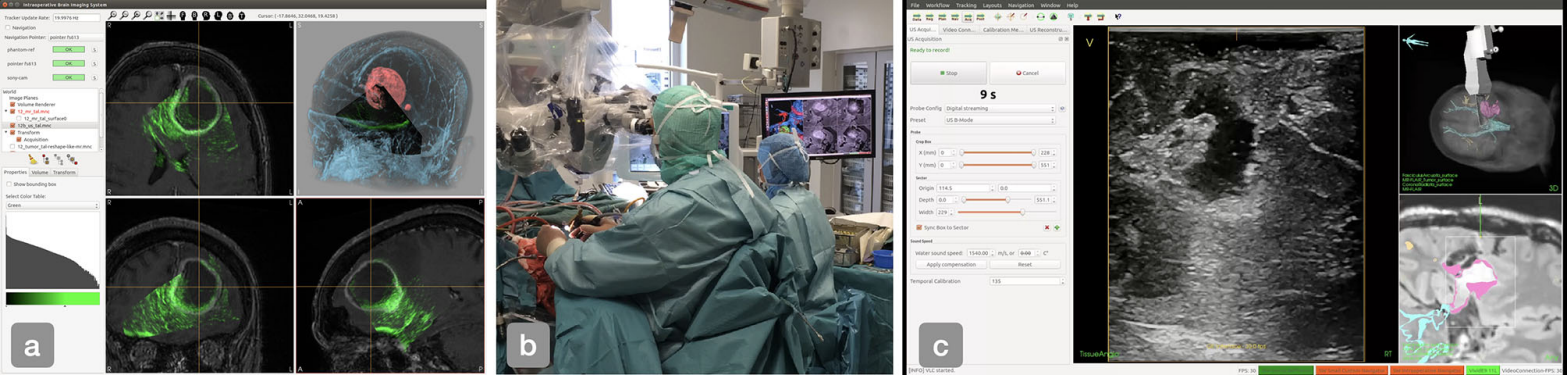
**(A)** The main interface of Ibis Neuronav where a case from the BITE databased was loaded, **(B)** iUS-based navigation in the OR using the CustusX system. **(C)** The main interface of CustusX during the acquisition of a US image sequence.

Ibis neuronav (3) is an open source surgical navigation platform originally developed at the Montreal Neurological Institute (MNI) in Canada. The platform provides all the basic functionality common to most commercial systems mentioned above. It can visualize a wide variety of 3D brain scans in the operating room (OR) and register the patient with the scans in order to display the location of tracked surgical tools in relation to the images displayed on a computer screen. The goal of the platform is to enable research projects that aim to improve upon existing commercial systems by providing an open implementation that can easily be modified and extended. The platform enables the validation of novel visualization, image processing and human-computer interaction methods in the OR and can be run in parallel with state of the art commercial solutions to enable comparison. Over the years, the Ibis system has been used for a variety of clinical applications such as brain tumor resection, neurovascular interventions (7), spine surgery (8), electrode implantation for deep brain stimulation (9), and the monitoring of epilepsy (10). However, the most notable contributions of the platform come from its ability to correct for brain shift based on intraoperative US imaging (11, 12) and its augmented reality visualization functionality (3). The system is able to capture and display images from a tracked US probe. It can acquire a sequence of such images and automatically reconstruct a volume from the individual slices. The computed US volume can then be used to compensate for mis-registration and tissue deformation by registering preoperative scans to the acquired US volume. The validation process for the brain shift correction functionality involved acquisition of tracked US sequences during a large number of neurosurgical procedures. Following these acquisitions, it rapidly became clear that the acquired US images together with other corresponding patient scans would be of interest for a wider community of medical image processing researchers, most of whom do not have access to neurosurgical operating rooms. The acquired data sets were thus made publicly available as the “Online database of clinical MR and ultrasound images of brain tumors” (BITE). We will describe this database in a later section. **Figure 1A**) shows an example of the main interface of Ibis Neuronav where the MR volume, iUS volume and iUS slices for one of the cases in the BITE database was loaded.

#### CustusX

The CustusX platform (2) in its current form was initiated in 2007 as the software platform for the Norwegian National Advisory Unit for Ultrasound and Image-Guided Therapy (USIGT)^3^. The platform is built on a number of open source libraries and toolkits and includes all the key components of a surgical navigation system. The system includes many of the same functionalities as Ibis Neuronav such as visualization of pre-operative MR/CT images in 2D and 3D, image-to-patient registration with various methods, tracking of ultrasound probes and surgical instruments, acquisition of 2D freehand ultrasound images, reconstruction of 3D ultrasound volumes and rigid MR-to-US registration for correction of brain shift. The main focus of the development has been intra-operative ultrasound imaging in different neurosurgical applications such as of brain tumor resection and neurovascular procedures procedures (13–15). The platform is also used in other clinical areas such as broncoscopy (16, 17), vascular surgery (18), and laparascopic surgery (19). **Figure 1B** shows how CustusX is being used in the OR for iUS-based navigation and **Figure 1C** presents an overview of the main interface during an iUS acquisition session.

#### Other Systems

A wide variety of open source guidance systems have been proposed over the years. Although several of them included some form of support for iUS, it has not been their main focus. These systems include 3D Slicer (20) and MITK (21). 3DSlicer, initiated and mainly developed by the Surgical Planning Laboratory (SPL) at Brigham and Women’s Hospital and Harvard Medical School in Boston, USA, is an open source software system for medical image processing and visualization. In recent years, 3DSlicer has been enhanced to support intraoperative navigation, ultrasound acquisition and advanced visualization (22). 3DSlicer is largely built on the same low-level libraries as Ibis and CustusX but has the additional advantage of being supported by a large and active community of developers and users worldwide. Although 3D Slicer has been used in various research prototypes of guidance systems based on US imaging, the setup of such systems from the distributed software components remains technically very challenging and the interface is not appropriate for use by clinicians. An important aspect to note however is that the SlicerIGT extension provides a mechanism to replace the user interface, enabling the creation of custom applications for specific clinical contexts and more appropriate for their use by clinicians.

The Medical Imaging Interaction Toolkit (MITK) (21, 23) is developed and maintained by the German Cancer Research Center (DKFZ) in Heidelberg, Germany. The platform was initiated more than 15 years ago as an open-source solution for image analysis, treatment planning and intervention support. The system has also been enhanced with support for real time ultrasound imaging (24). MITK has been used in applications such as robot-assisted ultrasound-guided radiation therapy (25) but not for ultrasound guided neurosurgery.

### Comparison of Existing Systems

Amongst the existing open source systems, we can distinguish two categories. Both Ibis Neuronav and CustusX are built specifically to be used as intraoperative navigation systems and are designed with a focus on iUS-based navigation, while 3D Slicer and MITK are general purpose imaging platforms that can be customized as navigation systems by installing extensions and/or modifying configuration scripts. Although the interfaces of Ibis and CustusX are designed by engineers and for engineers, they are simple enough to envision training clinicians to use them without technical assistance, but it is not the ideal scenario for a multi-center research projects. On the other hand, both 3D Slicer and MITK allow developers to completely overwrite the default user interface, which opens the possibility of developing a simplified, clinician-centered interface specialized for iUS-based navigation and intraoperative data acquisition. In the case of 3D Slicer, the interface can be overwritten with a simple Python script, which can greatly ease the development process. One important aspect to consider in choosing an open source system is the level of activity in the community of developers and users. On this aspect, 3D Slicer has the most active community by far, even though its developers spread their efforts over a much larger number of features. **Table 1** summarizes the differences between existing systems.

**Table 1:** Comparison of features provided by different open source navigation platforms in regard of acquisition, rendering and navigation using US imaging (NT, Non-trivial).

Feature	CustusX	IBIS	Slicer	MITK
Configurable 2D/3D graphic window layout	Yes	No	Yes	Yes
Comprenhensive 3D Transforms system	Yes	Yes	Yes	Yes
Overwrite user interface	No	No	Yes	Yes
2D Slice rendering	Yes	Yes	Yes	Yes
3D Surface rendering	Yes	Yes	Yes	Yes
Volume Rendering	Yes	Yes	Yes	Yes
Augmented Reality	No	Yes	No	No
US capture	Yes	Yes	NT	NT
3D Display of US slice	Yes	Yes	NT	NT
US Volume Reconstruction	Yes	Yes (GPU)	No	No
Linear MR-US or CT-US registration	Yes	Yes (GPU)	NT	NT
US probe calibration	NT	Yes	No	No
3D tools tracking	Yes	Yes	NT	NT

### Existing Databases

#### MNI BITE

The “Brain Images of Tumors for Evaluation” (BITE) database (5) is a publicly available dataset composed of MR and iUS images captured during 14 surgical procedures to remove brain tumors.^^4^^

All patients were operated at the Montreal Neurological Institute (MNI) by two different surgeons, and the iUS data was acquired using the IBIS Neuronav system described above. All patients presented with gliomas of varying grades. For each of the cases, the database contains a preoperative MR, multiple sequences of iUS and a postoperative MR. Here is a detailed description of the images:

- Preoperative scans: T1 weighted gadolinium-enhanced MR images acquired at 1.5T, with 1 mm slice thickness and a 0.5 mm in-plane resolution, except for one case that was acquired at 3T, with 1 mm slice thickness and 1 mm in-plane resolution.
- Postoperative scans: T1 weighted gadolinium-enhanced MR images acquired at 1.5T with 0.5 mm in-plane resolution, but have varying slice thicknesses (between 1 and 5 mm).
- iUS sequences: US images acquired from a Philips HDI 5000 scanner by capturing the analog video output. A P7-4MHz phased array transducer was used and set for a scanning depth of 6.5 or 8 cm, depending on the size and location of the tumor. The probe was tracked in 3D using an NDI Polaris optical tracking system. Each of the acquisitions consists of an approximately linear sweeps of 200–600 frames where the probe is moved at approximately 3 mm/s. In addition to the images themselves, the database contains a rigid transform obtained from the tracking system for every image. Each patient has multiple US sweeps and all of them, except for one, have sweeps pre- and post resection.

In addition to the raw data described above, the database contains the following processed/manually specified data for each case:

- A set of 10 homologous landmarks identified by a neuroradiologist that maps one pair of pre and post resection sweeps
- A US volume obtained by concatenating all the slices from a single iUS sweep.
- A set of between 19 and 37 homologous landmarks that maps preoperative MR and pre-resection US.
- The patient to preoperative MR transform obtained by aligning a set of homologous points identified on the scans prior to the operation and acquired on the patient using a tracked pointer during the operation.

Following the success of the BITE database, 25 more tumor cases were acquired to form the basis for a second version of the database. An initial set of 9 cases have already been released and described in (26). The new version of the database contains essentially the same information as the first version and has been complemented with preoperative FLAIR and T2 weighted MR scans when available. In addition to the various sets of homologous landmarks that can be used as ground truth for the registration of the different imaging modalities, the new version of the database contains a segmentation of the tumor based on all preoperative modalities available, namely the T2 tumor hyperintensities (edema), the enhancing tumor core and the non-enhancing tumor core. The complete database comprising all 25 cases is in preparation and will be released shortly.

#### RESECT

The “Retrospective evaluation of Cerebral Tumors” (RESECT) database (6) consists of MR and ultrasound images of 23 low grade gliomas that have been resected at St. Olavs University Hospital, Trondheim, Norway by one neurosurgeon. ^5^The database includes pre-operative contrast enhanced T1-weighted images, pre-operative FLAIR images and 3D ultrasound images acquired before, during and after tumor resection. More specifically, the database contains:

- Preoperative MR images: T1 weighted gadolinium (Gd)-enhanced and fluid attenuated inversion recovery (FLAIR) MR images acquired at 3T, both with 1 mm isotropic voxel size. For three patients, pre-operative images were acquired at 1.5T with a slice thickness of 1 mm and a 0.5 mm in-plane resolution for the Gd-enhanced T1 images and 1mm isotropic voxel size for the FLAIR images. The MR images include the patient registration transform.
- Intra-operative US images: US images acquired using the Sonowand Invite neuronavigation system (Sonowand AS, Trondheim, Norway) which includes a digitally integrated ultrasound scanner. A 12FLA-L linear transducer with a frequency range of 6–12 MHz was used. Depth and gain were adjusted depending on the size and location of the tumor. The probe was tracked in 3D using the NDI Polaris optical tracking system integrated in the Sonowand Invite system. The raw ultrasound data were reconstructed into 3D volumes using the reconstruction method included in the Sonowand Invite system. For all patients, the database includes one ultrasound volume acquired before resection (after opening of the dura), one ultrasound volume acquired during the resection and one ultrasound volume acquired after the resection.

In addition to the images, the database contains for each patient:

- Two sets of between 10 and 34 landmarks mapping the first (before resection) ultrasound volume and the second (during resection) and third (after resection) ultrasound volumes.
- Two sets of between 12 and 16 homologous landmarks mapping the pre-operative FLAIR volume to the first (before resection) ultrasound volume or the third (after resection) ultrasound volume.

The database does not contain any post-operative data or segmentations.

## Results—Impact of Existing Databases BITE and RESECT

Together, the BITE and RESECT databases have been downloaded more than 1,000 times and have enabled the publication of more than 110 (number of citations as of Oct 2020) research articles, which illustrates the need and interest from the research community. Most of theses articles concerned the development and evaluation of registration algorithms for correction of brain shift, which is in line with the initial intended use. The public availability of the databases has enabled the development of alternative approaches to brain shift correction by groups with different expertise. The availability of BITE and RESECT has also enabled research on previously unforeseen applications.

### MR-US and US-US Registration

The main purpose of the RESECT and BITE database was to provide a public dataset with real clinical data for evaluation of MR-US and US-US registration algorithms for correction of brain shift. These methods include the use of different similarity metrics (27–33), segmentation-based registration (34), and deep learning (35–37). So far, the conventional registration methods using similarity metrics well adapted to ultrasound images have proven to be the most successful (38). The work of Machado et al. (32) is particularly interesting in the way it focuses on robustness, and not only accuracy, of the registration and validate their results on BITE and RESECT in addition to MIBS, a proprietary database from Brigham and Women’s Hospital. The article highlights the need for larger publicly available datasets collected from different centers as robustness is key to the adoption of iUS-based correction of brain shift in the standard of care.

As the databases do not contain any segmentations of tumors or other structures, segmentation-based approaches require the authors to perform their own segmentations to obtain a ground truth. This is challenging and time consuming, and limits the development of such methods. Several groups have tested deep learning approaches. However, the databases only contains data from 46 patients, which is probably insufficient for these methods to perform well and presents a high risk of overfitting. The RESECT database has also enabled the organization of registration challenges (CuRIOUS 2018 and 2019, Learn2Reg 2020) in conjunction with the International Conference on Medical Image Computing and Computer Assisted Interventions (MICCAI) where a number of different approaches have been benchmarked. The methods and results from the CuRIOUS 2018 challenge are summarized in (38).

The provided images have also been used together with other available databases for evaluation of registration metrics. Luo et al. analyzed the validity and distribution of landmark points provided with both RESECT and BITE (39). The paper highlights the need for a mechanism that allows users of such databases to contribute back improved data generated by users, in this case an improved version of landmark positions for points with a potentially high fiducial localization error (FLE).

### Segmentation

One of the most promising avenues to facilitate the interpretation of iUS images is to automatically segment anatomical structures to enhance the visualized image with annotations. Several projects have used the data in BITE and RESECT to develop and validate segmentation methods for different structures. Canalini et al. used data from both databases to segment sulci and falx cerebri in US volumes using a convolutional neural network (CNN) (34). The segmented structures were then used to register together multiple US acquisitions at different stage of the resection and study the evolution of the procedure. It is interesting to note that for this paper, a manual segmentation of the sulci and falx cerebri has been performed using a custom tool built into the MevisLab platform^6^. Unfortunately, the current distribution systems for BITE and RESECT do not include functionality to contribute back this kind of annotation, which might be useful for other research groups. In many situations, it is important for a surgeon to determine the location of the boundary between gray and white matter. In Demiray et al., (40), authors use a CNN to perform the segmentation of gray and white matter from the 3D iUS images of the RESECT database. Given the difficulty for a clinician to manually segment those structures from iUS images alone, a ground truth segmentation was obtained automatically from the co-registered MR images distributed with RESECT. Another structure that has been segmented from the RESECT data is the resection cavity. This information can not only facilitate modeling of tissue deformation during surgery, but it can also help to identify residual tumor tissue that needs to be resected in order to prevent the recurrence of a tumor. Carton et al. used both BITE and RESECT to train a U-NET based model to perform surgical resection cavity segmentation in US images (41). For their work, they produced a manual segmentation of the resection cavity in US images. Again, this sort of annotation of the data could be beneficial to many other research teams if it could be contributed back to the databases. A more difficult, but very important problem in US image processing is that of segmenting brain tumors. This type of tissue can have a variety appearance depending on tumor type. Golb and al. used the RESECT database to develop and validate a tumor segmentation method based on US images (42). Maani et al. use the MR images from the BITE database to validate their tissue classification method based on volumetric texture analysis (43).

### Other Applications

The availability of the open databases has also enabled research in other areas related to US guided neurosurgery. One example is the work of Sagheer et al. (44) who used the BITE database to validate their US image denoising algorithm. Other examples include a US probe calibration method (45) where the authors used the BITE database in the validation process, simulation of 2D US from 3D MR (46) and sorting of DICOM images (47). Open image databases such as BITE and RESECT can also be used for more clinically oriented research. Petrecca et al. (48) used images from BITE to analyze the patterns of recurrence of glioblastomas.

## Discussion

In the previous sections, we have demonstrated that the public availability of US image databases such as BITE and RESECT has had an impact far beyond the initial intended purpose of the data collection process. Despite the relatively limited number of cases released in the databases, their availability has enabled significant progress in the development of algorithms that increase the usefulness and accuracy of iUS-based neuronavigation. First and foremost, a wide variety of MR-US registration algorithms have been proposed to compensate for brain shift and restore navigation accuracy at any moment during an operation. The databases have also enabled early successes in the development of multiple US image segmentation algorithms and a variety of other algorithms that are key to increasing the usefulness of US imaging in neurosurgery. In this section, we review the limitations of existing data sets, we show the importance of open source software to accelerate and standardize the acquisition process and we lay out a plan for the next generation of publicly available US imaging data.

### Current Limitations and Future Needs for Technical Development and Data Collection

#### Registration

Despite the great advances in the iUS-based correction of brain shift we have seen with the previous generation of databases, the technology has yet to be adopted in commercial navigation systems and to be used as the standard of care. As a result, surgeons tend to simply stop relying on navigation systems once tissues have moved and deformed beyond a certain threshold. One of the reasons for this slow adoption by the major commercial systems is the lack of scientific evidence on the robustness of existing registration algorithms. Several questions remain as to the quantity, quality and acquisition protocol that will guarantee a stable and accurate correction of brain shift in every case. To be able to validate the robustness of MR-iUS registration algorithms, future data collection efforts will need to include a much larger number of patients from several centers and clinicians with different levels of training. Such data sets will not only enable the validation of the robustness of registration algorithms but will also allow for the development of iUS image acquisition guides that will help ensure that the quality and quantity of data is sufficient to result in a stable registration every time.

#### Segmentation

With the recent developments in machine learning, we have started to see some progress in the area of segmentation of US images, a problem that was previously considered notoriously difficult. Segmented structures can be used to either improve image registration and further correct for brain shift, or improve visualization by highlighting different structures in real-time. For deep learning methods to perform well, large amounts of data are needed for training and testing. The number of cases needed depends on the difficulty of the segmentation problem (the image contrast between the structure to be segmented and its surroundings), the morphological variability of the structure to be segmented and the required accuracy. The training dataset needs to cover the full spectrum of anatomical and pathological variability in addition to differences due to scanners type, settings, and operators. Examples of segmentation of US using deep learning in other clinical areas are 1) Smistad et al. (49), where the authors segmented nerves for guiding regional anesthesia using data from 49 subjects and, 2) Anas et al. (50) where they segmented the prostate for targeted biopsies using data from 18 patients. These and other studies show promising results, but the number of included subjects is still limited. It is unlikely that the variability is fully covered, and the external validity of the results might therefore be limited. So far, there are no studies on the amount of ultrasound data required to detect or segment brain structures using deep learning methods. However, a study of deep learning for automatic segmentation in echocardiography suggests that 2D ultrasound images from 250 patients are needed for accurate segmentation of the left ventricle of the heart (51). As part of this study, a dataset with images from 500 patients, including expert annotations, was made publicly available (CAMUS data set) and it is now widely used for deep learning in the field of echocardiography (55 citations since 2019 as of Oct 2020). Even in cases with poor image quality in echocardiography, the image contrast is higher than in many ultrasound images of brain tumors. For segmentation of brain tumors, where contrast might be low and high accuracy is needed, it is reasonable to expect that at least a comparable number of cases is needed. For other relevant brain structures such as the lateral ventricles, the sulci and the falx that usually have higher contrast, fewer cases might be needed. These structures are important landmarks for the neurosurgeons, and automatic delineation would greatly ease the interpretation of the ultrasound images and thus address one of the main hurdles for widespread use of ultrasound in neurosurgery. However, as ultrasound images do not typically cover the entire brain, not all patient datasets will contain information about all structures. To collect several hundred data sets from brain tumor patients, multi-center data collection is the only possible way forward.

#### Visualization

One of the usability problems of iUS most often reported by neurosurgeons is the difficulty to simultaneously acquire and visualize the images. When imaging other parts of the anatomy such as abdominal organs, experienced ultrasonographers typically look at the screen of the scanner to locate the anatomy of interest and rely mostly on their sense of proprioception to physically position the US probe on the skin of the patient and maintain the appropriate amount of pressure. Imaging the brain is different because acquisition has to be performed on the very limited surface of the dura or cerebral cortex exposed by a craniotomy and almost no pressure can be applied to the delicate tissues of the brain. Thus, the surgeon’s attention has to remain on the surgical field at all time, which results in an acquisition being performed without visual feedback first, and then visualized. This can force a surgeon to perform multiple cycles of acquisition and visualization to obtain the desired information. Several groups have proposed the use of augmented reality (AR) to solve this problem. The technology of AR enables the display of US images in-situ, allowing neurosurgeons to visualize the images being acquired while maintaining their attention on the surface of the operating field. So far, most of the research on neurosurgical AR has focused on the integration of hardware and software components required to accurately overlay virtual content such as US images, with a live view of the operating field. However, one of the main obstacles to the adoption of AR remain the various depth perception problems generated by the live mix of real and virtual content. In order to accelerate research on neurosurgical AR and in particular on live in-situ display of iUS images, a future intraoperative image database should include live video of the operating field allowing researchers working on the improvement of AR rendering techniques to evaluate their method on realistic models of the operating field. Images can be captured from a variety of sensors. For example, the Ibis Neuronav system includes a built-in module to produce AR images from a surgical microscope that has the ability to record live images (3). An extension of Ibis called MARIN adds similar capabilities to produce AR image from a tablet computer (52).

### Interrelationship of Software and Data

The development of the Ibis Neuronav system and the following acquisition of the BITE dataset exemplify the interrelationship between software and data and the value of an open source surgical navigation system in the data acquisition process and in the advancement of iUS based surgical navigation. The version of the Ibis system used to acquire iUS images for the BITE database included only rudimentary functionality to reconstruct a 3D volume for 2D iUS slices, a process which took on average 10 min (5). Similarly, Ibis implemented a well-established method to linearly register an iUS volume with preoperative MR scans (53) to correct for brain shift, which also ran in approximately 10 min. Overall, the process of correcting for the patient-to-image misregistration took on average 20 min once the surgeon had finished a US image sweep. Given such delays, it was difficult to convince clinicians of the benefits of the method. It also made the data acquisition process error prone as once the reconstruction and registration process finished, it was already too late to perform a second acquisition if it turned out the first one did not contain enough information to obtain a reliable registration. The release of the first BITE database stimulated the research on registration algorithms that were more appropriate for MR-US registration. One of the most robust methods proposed was based on the alignment of image gradient (33) and provided a GPU implementation that authors agreed to contribute to the source code of Ibis as an extension of the platform, together with a GPU implementation of iUS volume reconstruction. The new volume registration algorithm ran in under 5 s, while MR-iUS registration took under 1 s (3). The improved system generated much more interest on the part of the clinicians as they were able to appreciate the results of the registration correction algorithm as soon as they finished acquisition. It also provided instant feedback on the quality of the acquisition, allowing them to improve faster on their acquisition skills, resulting in an increased pace of data acquisition. As a result, a second version of the BITE database (soon to be released), containing double the number of cases of the first one (25), has been acquired in a shorter time frame.

Similarly, a GPU implementation of a MR-iUS registration algorithm (54) was integrated into CustusX and validated intra-operatively in a series of 13 brain tumor cases (14). This method provided registration results within a few seconds and ran seamlessly within the CustusX software.

While research platforms such as Ibis and CustusX have been useful and successful in a large number of research projects, they might not be suitable for a large scale data collection effort. The systems have clearly improved over the years, but they are still highly complex. The use of these systems requires the presence of a dedicated technical researcher in the operating room during every single procedure in order to ensure the correct functioning of the system. The complexity of their numerous options and configurations combined with a sub-optimal user interfaces makes these systems unsuited for clinical users, and thus unsuited for efficient collection of high volumes of intra-operative data from hundreds and possibly thousands of patients.

### Requirements and Architecture for the Software and Database of the Future

In order to efficiently collect a large number of patient cases, a simplified software solution better adapted to brain tumor surgery is required. The software needs to include all the basic components of a neuronavigation system like Ibis and CustusX, but the user-interface should be redesigned and adapted with clinical users in mind. This means a highly intuitive interface with only the essential components, fewer buttons, menus, and configurations than the existing systems.

The user-interface should be designed to provide the right information at the right time and to fit into the clinical workflow in neurosurgery. The system should be adapted to run in parallel with commercial navigation systems without imposing additional preparation or manipulation by the surgeon. More specifically, the following steps should be performed simultaneously for both systems:

- Calibration of tools such as navigation pointer and ultrasound probe
- Pre-operative image-to-patient registration
- Ultrasound acquisition (when available in commercial systems)
- Pointer-based navigation

In addition to replicating the functionality of the commercial systems, the proposed platform should provide a fast mechanism to reconstruct a 3D volume from a sequence of iUS images and register preoperative scans to the reconstructed volume to correct for brain shift. From a developer’s point of view, it should be straightforward to replace the default volume reconstruction and registration algorithm with newly developed methods as they become available.

The proposed system should also include improved visualization capabilities to provide visual feedback during the acquisition of iUS images. It should be capable of displaying the result of real-time automatic segmentation of arbitrary structures during the acquisition. For developers, a simple software development interface should allow to easily integrate new segmentation algorithms as they become available, without having to deal with the visualization aspect that would be natively supported by the system. The proposed system should also include modules to produce AR views of the operating field in order to provide visual feedback to the surgeons during the acquisition process, while allowing them to keep their attention focused on the operating field. The additional data captured during the AR visualization sequences should be automatically recorded. For example, when creating AR images with a tracked tablet computer like it is done with the MARIN system mentioned earlier, live video and tablet tracking transforms should be recorded to enable the improvement of AR rendering techniques in the laboratory.

The proposed system should automate the process to transfer the collected datasets from different centers to a common cloud-based database. In addition to various national and local solutions, large-scale international solutions for such cloud-based systems include:

- The “Imaging Data Commons” (https://imagingdatacommons.github.io/)
- ELIXIR Data Platform (https://elixir-europe.org/platforms/data)
- HRIC (55)

This infrastructure must ensure data storage and processing within the relevant ethical, legal, and regulatory frameworks such as EU’s General Data Protection Regulation (GDPR). Following data collection, all datasets should be automatically anonymized and controlled for completeness and quality. The format of the database for each case should be flexible enough to take into account differences in imaging protocols at different centers. For example, certain centers might not acquire FLAIR images in their standard protocol for brain tumor cases, so the database should support uploading data without this type of image. The database should allow users to filter cases based on the information available. The proposed database should also support the addition of new types of data that were not planned for during development.

In order to be used for image analysis and machine learning, different levels of data annotation is needed. Annotation needs to be performed by clinical experts through the use of dedicated software solutions such as a secure web-based platform for example. The images, video, position information etc. should be made available in widely used open formats directly compatible with the most commonly used open source imaging and machine learning toolkits and libraries such as TensorFlow and Keras.

## Conclusion

In this paper, we have reviewed the impact that the open source neuronavigation platforms Ibis Neuronav and CustusX and their associated databases BITE and RESECT have had on the progress iUS-based navigation. We have particularly emphasized how the synergy between open source software and publicly available data has contributed to accelerate this progress. Building on these successes, we have proposed to combine the effort of multiple research groups to build a simplified and improved combination of open software and tumor case database that is likely to enable gathering the large amounts of data needed to train new machine learning models to improve iUS-based navigation. The main goal of this data collection effort is to provide the international research community with high quality data. This will accelerate research in the field, especially among research groups who do not have the possibility to collect their own data but rely on publicly available datasets. With more research groups working actively in the field, the development of new tools and methods will also accelerate. New, innovative, and well validated tools can then be included in the open software platform which will enhance the usefulness for clinician and thus accelerate data collection through an increased rate of new cases. This cycle can thus be a positive self-reinforcing process that will lead to more robust and accurate tools, acceleration of the translation from the laboratory to industry and more accurate, safer, and more minimally invasive procedures for patients.

## Data Availability Statement

The original contributions presented in the study are included in the article/supplementary material; further inquiries can be directed to the corresponding author.

## Author Contributions

IR and SD drafted the manuscript. All authors contributed to the article and approved the submitted version.

## Funding

This work has received funding from the Norwegian National Advisory Unit for Ultrasound and Image-Guided Therapy (usigt.org) as well as from the Natural Sciences and Engineering Research Council of Canada (CHRP 385864) and the Canadian Institute for Health Research (MOP-97820).

## Conflict of Interest

The authors declare that the research was conducted in the absence of any commercial or financial relationships that could be construed as a potential conflict of interest.

## Footnotes

1 https://www.brainlab.com/surgery-products/overview-neurosurgery-products/intraoperative-ultrasound/

2 https://github.com/IGSIO

3 https://usigt.org

4 The BITE database is avilable at http://nist.mni.mcgill.ca/?page *_i_d*= 672

5 The RESECT database is available at https://archive.norstore.no

6 https://www.mevislab.de/
